# Biophysical studies of cholesterol effects on chromatin[S]

**DOI:** 10.1194/jlr.M074997

**Published:** 2017-04-28

**Authors:** Isabel T. G. Silva, Vinícius Fernandes, Caio Souza, Werner Treptow, Guilherme M. Santos

**Affiliations:** Laboratório de Farmacologia Molecular, Departamento de Farmácia,* Universidade de Brasília, Brasília, Brazil; Laboratório de Biologia Teórica e Computacional, Departamento de Biologia Celular,† Universidade de Brasília, Brasília, Brazil

**Keywords:** lipids, physical biochemistry, molecular biology

## Abstract

Changes in chromatin structure regulate gene expression and genome maintenance. Molecules that bind to the nucleosome, the complex of DNA and histone proteins, are key modulators of chromatin structure. Previous work indicated that cholesterol, a ubiquitous cellular lipid, may bind to chromatin in vivo, suggesting a potential function for lipids in modulating chromatin architecture. However, the molecular mechanisms of cholesterol’s action on chromatin structure have remained unclear. Here, we explored the biophysical impact of cholesterol on nucleosome and chromatin fibers reconstituted in vitro and characterized in silico the cholesterol binding to the nucleosome. Our findings support that cholesterol assists 10 and 30 nm chromatin formation and induces folding of long chromatin fibers as a result of direct interaction of the cholesterol to six nucleosomal binding sites.

Chromatin is a macromolecular complex composed of distinct molecules. The nucleosome is the repetitive basic unit of chromatin. Nucleosomes arise from the precise stoichiometric interaction of proteins and DNA. Positively charged histone proteins interact with the DNA and provide an optimal strategy to functionally pack the genetic code inside a cell.

Changes in chromatin architecture are decisive for regulating the access of transcription factors, coregulators, and the basic transcription machinery to specific enhancers in target genes [reviewed in (1)]. Thus, chromatin state defines the functional genomic regulation.

The influence of different ions, such as of divalent cations Mg^2+^ or Ca^2+^, on the nucleosome and chromatin structure has been well-characterized in vitro (2). Under low-salt conditions in vitro, chromatin adopts an open conformation known as “beads on a string” or the 10 nm fiber. In a physiological ionic environment, with the presence of Mg^2+^ and the correct linker histone stoichiometry, the chromatin can fold to form a compacted chromatin structure, the 30 nm fiber (3). However, the impact of chromatin folding states on gene expression and phenotypic outcomes is difficult to predict due to the dynamic movement of the chromatin fibers in the nuclear environment. Furthermore, the full complement of nuclear molecules that affect chromatin structure and affect fundamental processes in the cell, such as transcription and DNA repair, is still unknown (4).

Although chromatin structural and functional states are largely defined by nucleosome binding proteins (NBPs) and the state of the histone H4 tail (5), the nuclear environment comprises several small molecules that may directly bind to chromatin [e.g., magnesium (6) and lipids (7)]. Conceivably, all these substances may have a considerable impact on chromatin architecture.

The characterization of endonuclear lipids has motivated a growing number of studies to hypothesize that this class of molecules may have an important and functional role on chromatin structure and gene expression (8).

Cholesterol is a ubiquitous cellular lipid, which is essential for steroid hormone production, the structural integrity of cellular membranes, and cell growth. Seminal studies in rats have shown that labeled cholesterol administered by gavage was detected within 2 h in the nuclei, associated with chromatin fibers purified from rats (9). Moreover, binding assays suggested that cholesterol binds to chromatin through one or more proteins (10). Later studies increased the understanding of the mechanisms of interaction between chromatin and small lipids and suggested that small lipid molecules, such as cholesterol and short-chain fatty acids, can regulate important epigenetic modifications that lead to chromatin structure changes (11). It has also been observed that the phosphoinositide pathway and changes in its nuclear profile may have a direct role in modulating chromatin structure (12).

Herein, motivated by the biophysical and biochemical aspects of chromatin interactions, we investigated the mechanism of cholesterol action on chromatin structure. In vitro, low concentration of cholesterol assisted 10 and 30 nm fibers and mononucleosome formation. Moreover, micrococcal nuclease (MNase) digestion, which targets nucleosome-free regions of long chromatin fibers, was inhibited in the presence of cholesterol. Finally, we used docking and molecular dynamics (MD) simulations to show that cholesterol interacts with nucleosomes through multiple binding sites.

## METHODS

### In vitro chromatin and mononucleosome reconstitution

The histone octamer (HO) and linker histone (H5) were purified from chicken erythrocyte nuclei as described in Ref. 13. To form mononucleosomes and long chromatin fibers, we used respectively, short DNA (167 bp) and long array (177 bp repeated 36 times) of 601 sequence (14). Titration of HO was performed to determine the saturation point, as described in Ref. 13. Competitor DNA was added only to form long chromatin fibers. Folding of nucleosomal arrays was carried out by dialysis into 1 mM MgCl_2_. Soluble cholesterol (S5442; Sigma) was added to the binding buffer or after dialysis when fibers or mononucleosomes were already formed. The analyses of reconstitution and folding were verified by electrophoresis in native agarose gels (0.8%) for long chromatin fibers and native bis-acrylamide gels (6%) for mononucleosomes. Electrophoresis was carried out with 0.2× TBE [18 mM Tris-borate (pH 8) and 0.4 mM EDTA) electrophoresis buffer at 20 mA. For folded chromatin, the samples were first fixed with 0.1% (v/v) glutaraldehyde on ice for 20 min. Densitometry was performed using ImageJ (National Institutes of Health, Bethesda, MD) version 1.49.

### MNase assay

Reconstituted long chromatin fibers (60 nM), in presence or absence of soluble cholesterol, were digested for 8 min with 0.08 u of MNase (New England Biolabs) at 37°C. Aliquots of samples were collected at 0, 1, 2, 4, and 8 min of digestion and stopped with 10 mM EDTA (pH 8.0). The digestion was analyzed by electrophoresis in native agarose gels (0.8%), carried out with 0.2× TBE electrophoresis buffer at 20 mA.

### Compaction of long chromatin fibers induced by Mg^2+^

Chromatin compaction by Mg^2+^ was adapted from Rhodes laboratory protocol (13). Briefly, long chromatin fibers were incubated with increasing concentrations of soluble cholesterol (1, 10, and 100 μM final) or labeled cholesterol (0.5 and 1 μM final) [TopFluor® cholesterol, 23-(dipyrrometheneboron difluoride)-24-norcholesterol; Avanti Polar Lipids] on ice for 30 min (water was used as control). Next, 4.5 mM MgCl_2_ were added and incubated for 15 min on ice. The compacted fibers were pelleted by centrifugation at 15,493 *g* for 15 min (4°C). The supernatants and pellets were verified by electrophoresis in native 0.8% agarose gels, carried out with 0.2× TBE electrophoresis buffer at 20 mA. Typhoon 9200 was used with 532 nm green laser excitation to visualize cholesterol fluorescence.

### Computational methods

Atomistic models were built using high-resolution X-ray structure of the nucleosome core particle (NCP), Protein Data Bank code 1KX5 (15), and cholesterol, Protein Data Bank code 1N83 (16). Following the strategy adopted in our previous studies on ligand binding to proteins (17), AutoDock Vina (18) was used to probe the binding of the cholesterol molecule against 120 NCP independent structures to account for receptor flexibility. These structures were extracted from the last 6 ns of ∼20 ns equilibration simulation of the NCP system. All docking solutions were clustered into different groups based on distance. Six putative binding sites for the cholesterol molecule were selected based on the protein region they were bound to. Two systems were then simulated for 200 ns in the presence of three cholesterol molecules representing the most stable conformation found at each binding site. Sites s1, s4, and s6 were simulated in one system, and sites s2, s3, and s5 were simulated in another one. Energy averages were sampled from these simulations in order to compute the binding constants for each cholesterol molecule by the linear interaction energy method (19, 20) (supplemental Fig. S6). All simulations were performed with the program, Nanoscale Molecular Dynamics (NAMD) (21), using the CHARMM 36 force field (22, 23) and TIP3 water model (24). The particle-mesh Ewald (PME) method (25) was employed on the electrostatic calculations and nonbonded interactions were cut off at 11 Å. All systems were simulated at 300 K, 1 atm, and with a 2 fs time step.

All images created and computational analyses were performed using visual MD (26). More details are provided as supplemental material.

## RESULTS

### Cholesterol assists mononucleosome and chromatin fiber formation in vitro

To better understand the effects of cholesterol on chromatin, we reconstituted long chromatin fibers and mononucleosomes in vitro using HOs purified from chicken erythrocytes (supplemental Fig. S1A) on a 601 bp DNA array. As a quality control of the chromatin reconstitution system, we analyzed open and compacted fibers by electron microscopy showing that this system was successful (supplemental Fig. S1B).

**Fig. 1.**
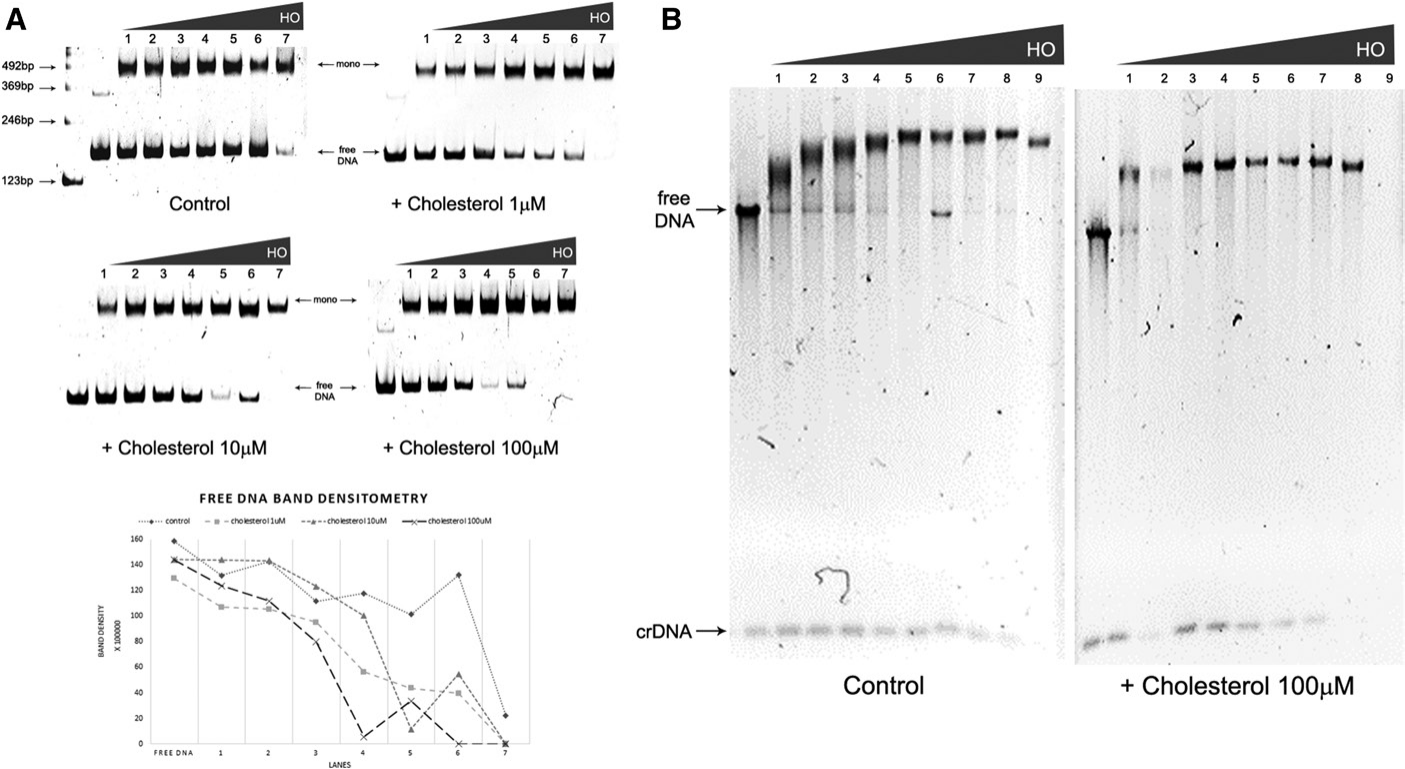
Effect of cholesterol on nucleosomes and chromatin fibers reconstituted in vitro. A: Mononucleosome formation: Chicken HO titration (1 = 1.3 μM; 2 = 1.5 μM; 3 = 1.8 μM; 4 = 2.0 μM; 5 = 2.2 μM; 6 = 2.5 μM; 7 = 2.7 μM) in the absence (control) or presence of increasing cholesterol concentration (1, 10, and 100 μM) analyzed by EMSA in acrylamide gel 6%. DNA: 167.1. Gels were run under the same experimental conditions; free DNA band densitometry graph of upper gels; B: Chromatin fiber formation: Chicken HO titration (1 = 2.0 μM; 2 = 2.2 μM; 3 = 2.5 μM; 4 = 2.7 μM; 5 = 2.9 μM; 6 = 3.1 μM; 7 = 3.4 μM; 8 = 4.0 μM; 9 = 4.7 μM) in the absence (control) or presence of cholesterol (100 μM), analyzed by EMSA in agarose gel 0.8%. Array: 177.36. Gels run under the same experimental conditions. Legend: crDNA, competitor DNA. All gels are representative of three separate experiments.

We used soluble cholesterol (SintheChol® NS0 supplement; Sigma) with proven physiological relevance (27, 28). Initially, we titrated three different concentrations of cholesterol during mononucleosome reconstitution (1, 10, and 100 μM) (**Fig. 1A**). When cholesterol was present, the HO concentration needed for DNA saturation was smaller than that needed for reconstitution without cholesterol (Fig. 1A). We observed that cholesterol, at final concentration at 1 μM, led to earlier DNA saturation by HO binding (Fig. 1A), as observed by the formation of mononucleosomes and loss of naked DNA. Pronounced cholesterol effects were also observed at 100 μM final cholesterol concentration. Under those conditions, addition of 2.0 μM (Fig. 1A, lane 4) HO was sufficient for complete DNA saturation. This effect is graphically represented in the free DNA band densitometry, showing the faster decrease of the DNA band in the presence of cholesterol. The same effect is demonstrated in another representative gel, presented in the supplemental material (supplemental Fig. S1C).

A similar phenomenon was observed when cholesterol was present during the long fiber reconstitution in vitro. In this case, DNA saturation was reached at lower HO concentrations. In the presence of cholesterol, the array 177.36 was saturated at 2.0 μM HO (Fig. 1B, lane 1) compared with control samples that were saturated at 2.9 μM HO (Fig. 1B, lane 5). Detailed analysis of HO titration on DNA also revealed DNA saturation at lower HO concentration in the presence of cholesterol (supplemental Fig. S1D, lanes 1–3). This result is made clearer by the formation of an unstructured material (faster migration in the gel and precipitation) at 2.7–3.4 μM HO (supplemental Fig. S1D, lanes 11–13) compared with control samples at the same HO concentration.

### Cholesterol favors chromatin folding in vitro

**Fig. 2.**
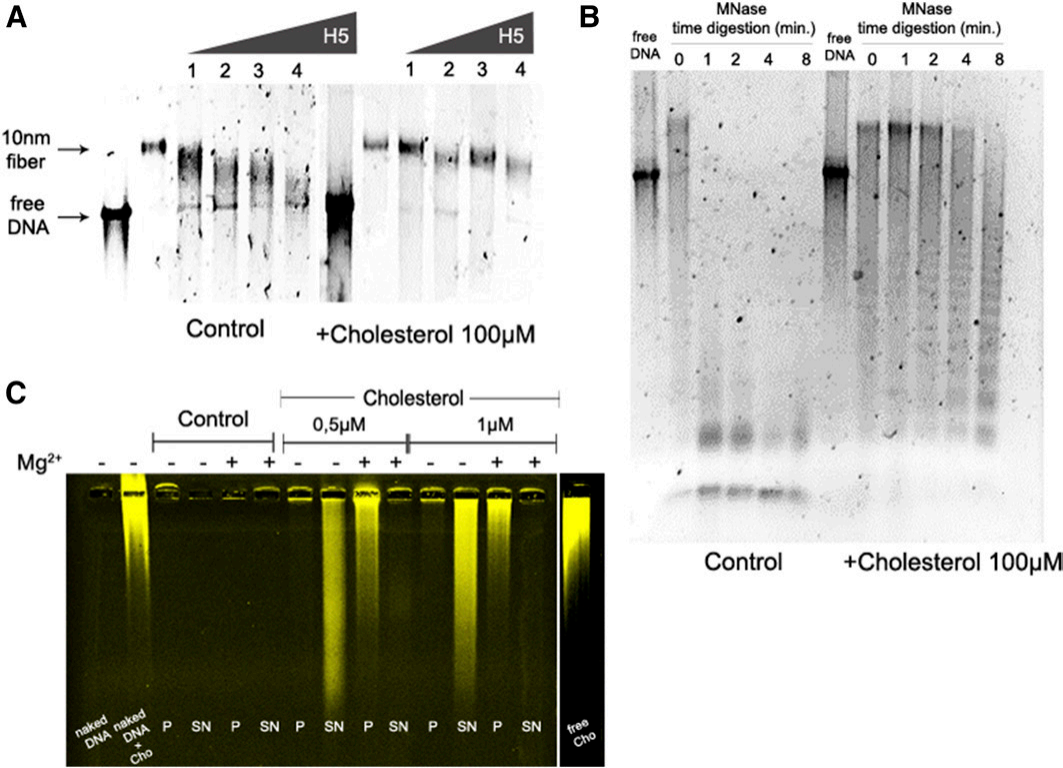
Cholesterol favors chromatin compaction in vitro. A: Compacted chromatin fiber formation: Titration of H5 [linker histone (1 = 1.5 μM; 2 = 3.0 μM; 3 = 4.6 μM; 4 = 6.2 μM)] on long chromatin fibers reconstituted in the absence (control) or presence of 100 μM cholesterol, analyzed by EMSA in agarose gel 0.8%. Array: 177.36. Gels were run under the same experimental conditions. B: Chromatin fiber digestion: MNase digestion of 10 nm fibers in the presence or absence of 100 μM cholesterol. Agarose gel 0.8%. Array: 177.36; C: Chromatin compaction assay: Compaction of 10 nm fibers induced by MgCl^2+^ in the absence (control) or presence of labeled cholesterol (0.5 or 1 μM), analyzed by EMSA in agarose gel 0.8%. Array: 177.36. Pellets (P), indicates compacted fibers; supernatant (SN), indicates open fibers; Cho, cholesterol. All gels are representative of three separate experiments.

Because open chromatin fibers may behave differently from compacted fibers when exposed to cholesterol, we next evaluated the effect of cholesterol on in vitro reconstituted 30 nm fibers with purified linker histones (H5) from chicken erythrocytes (supplemental Fig. S1A) in the presence of Mg^2+^, which is a divalent ion with characterized properties to induce chromatin folding (3). Similarly to the previous results with 10 nm fibers, we observed that cholesterol also assisted the formation of the 30 nm fiber in vitro (**Fig. 2A**). Control samples showed diffuse bands in the native gel, indicating a high degree of heterogeneity in the system; the reconstitution performed in the presence of cholesterol showed sharper bands, suggesting a more homogenous compacted chromatin.

Because we observed that chromatin folding was facilitated by cholesterol, we wondered whether the effect of cholesterol on chromatin formation might have structural consequences. To evaluate this possibility, we performed MNase digestion of chromatin fibers formed in the presence or absence of cholesterol. We found that MNase digestion of long chromatin fibers was inhibited in the presence 100 μM of cholesterol (Fig. 2B). Importantly, the presence of cholesterol did not affect MNase digestion of free DNA (supplemental Fig. S1F); in fact, it improved the DNA digestion. These results are consistent with a model in which cholesterol affects chromatin formation by increasing chromatin compaction.

Next, we sought to verify whether cholesterol exerts its effects by directly binding to chromatin and inducing compaction of long chromatin fibers. It is well-described that chromatin compaction is induced by cations such as Mg^2+^, which is known for promoting nucleosome-nucleosome interactions (6). Long chromatin fibers treated with 0.5–1.0 μM of labeled cholesterol were exposed to Mg^2+^ to induce compaction. Strikingly, cholesterol bound avidly to open or compacted chromatin fibers. In low concentrations (0.5 and 1 μM), labeled fibers moved from the supernatant to the pellet in the presence of Mg^2+^ (Fig. 2C). Labeled compacted fibers induced by Mg^2+^ showed decreased electrophoretic mobility when compared with open fibers, with the majority of compacted fibers being retained in the well. The presence of extensive smears in the gel was noticeable, which was due to the presence of free labeled cholesterol dissociated from the chromatin fibers. Due to technical limitations, we did not perform analytical ultracentrifugation with the long chromatin fibers in the presence of cholesterol to verify the precise degree of compaction.

It was not trivial to define the optimal concentration of cholesterol to be used in our in vitro system that would be physiologically relevant. The physiological concentration of cholesterol in the blood is approximately 5 mM. However, the apparent *K_d_* of cholesterol bound to chromatin was estimated to be approximately 1 nM (10). Herein, our in vitro system rendered chromatin fibers at a final concentration of 60 nM and mononucleosomes at 0.9 μM, and the cholesterol used was at 100 μM. This concentration gave approximately 1,600 cholesterol molecules per chromatin fiber with 36 nucleosomes, providing 45 cholesterol molecules per nucleosome. Considering that approximately 3,000 water molecules were found in the atomic structure of a nucleosome (29), with 121 molecules being important for HO interactions with the DNA, we believe that 45 cholesterol molecules per one nucleosome is stoichiometrically reasonable.

### Cholesterol interacts with nucleosomes through multiple binding sites

Collectively, our findings indicate that cholesterol plays an important role in chromatin structure in vitro, a result that may rely on cholesterol binding to nucleosomes. To evaluate this hypothesis, we applied docking and MD-based free-energy calculations to study binding of cholesterol at the atomic level. Specifically, we docked the ligand against an ensemble of equilibrated structures of nucleosomes, generated from tens of nanoseconds of MD simulation of the X-ray structure of the construct (29). Starting from cholesterol-bound structures, we carried out free energy calculations to resolve site-specific affinities of the ligand against the nucleosomes. The linear interaction energy method was used for that purpose (see the supplemental material).

**Fig. 3.**
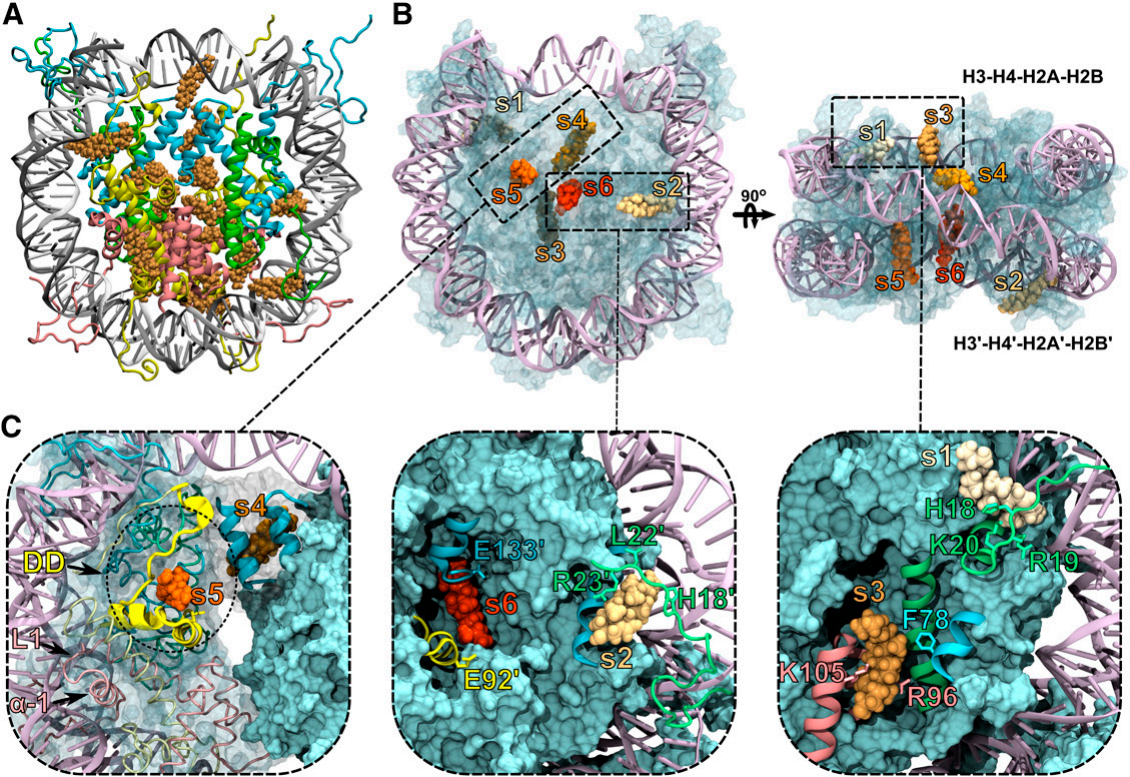
Cholesterol binds the nucleosome through multiple sites. A: Docking solutions for cholesterol (orange) against the NCP. B, C: Bound cholesterol at sites s1 through s6 along each of the histone units H3 and H3′ (blue), H4 and H4′ (green), H2A and H2A′ (yellow), H2B and H2B′ (pink). The docking domain (DD) and H2B loop 1 (L1) and helix 1 (α-1). Highlighted are amino acids of the acidic patch (E91′ and E92′ from H2A′), of the H4/H4′ tail (H18, R19, K20, L22, R23), and amino acids F78, E133′ from H3 and R96, K105 from H2B, which were previously reported to bind NBPs (5).

The docking results were clustered into 20 cholesterol groups at the protein-nucleosome surface (**Fig. 3A**). Interestingly, in six sites, the cholesterol lays very close to several amino acids, well-known to participate in the nucleosome structure and chromatin dynamics. Moreover, some cholesterol-contacting amino acids have also been correlated to nucleosome-nucleosome interactions or to nucleosome binding molecules. These interaction spots, hereafter called sites s1 through s6 (Fig. 3B, C), were selected for further MD and free energy calculations.

As detailed in supplemental Table S1, by binding to these multiple sites, cholesterol interacts with key nucleosome amino acids at the H4 tail, the acidic patch, and the docking domain in between the histone clusters H3-H4 and H2A-H2B (30).

After 200 ns of simulation, all six cholesterol molecules remained bound to the nucleosome under equilibrium conditions. As presented in supplemental Fig. S2, the nucleosome structure remained stable in its starting conformation throughout the simulation. In the presence of ligands, the root-mean-square deviation profile for the nucleosome converges to a plateau value of 2.0 Å, indicative of structural stability of the construct. Contrasting to the nucleosome structure, the bound ligand remains as flexible as in the free state in solution, which ensures a dynamic binding mode across the receptor binding sites (supplemental Fig. S3).

The close interaction between cholesterol and nucleosome suggests favorable binding affinities of the ligand to the receptor sites. To quantitatively evaluate this, we proceeded with a series of independent free energy calculations to estimate the per-site affinity of cholesterol to nucleosome. As shown in **Table 1**, ligand affinities are heterogeneous and span a large range of values (0.01–0.2 mM^−1^). For every site, the estimated absolute free energies favor cholesterol binding, rationalizing the stability of the molecular complex observed in the equilibrium MD simulations.

**TABLE 1.** Calculated values of binding free energies of cholesterol against the nucleosome

Sites	ΔG° (kcal·mol^−1^)	K (mM^−1^)
s1	−1.75 ± 0.18	0.0188
s2	−2.78 ± 0.16	0.1060
s3	−1.29 ± 0.17	0.0087
s4	−1.66 ± 0.17	0.0162
s5	−3.13 ± 0.16	0.1906
s6	−2.43 ± 0.16	0.0589

## DISCUSSION

Cholesterol, an abundant cellular lipid, is among the molecules interacting with eukaryotic chromatin in vivo. However, very little is known about the mechanism and biological role of cholesterol-chromatin interactions.

Herein, we showed, in vitro and in silico, that cholesterol has an anticipated role in modulating chromatin structure and provided atomistic insights into the impact of cholesterol on the nucleosome structure. Specifically, cholesterol appears to facilitate chromatin folding, verified by its effect on compacted chromatin fiber formation and by the inhibition of MNase digestion of long chromatin fibers. Complementary computational studies suggested direct molecular interaction of cholesterol with the nucleosome through six binding sites nearby important interacting regions for NBPs. Thus, our results support a model in which cholesterol induces chromatin formation and folding via direct interaction with nucleosomes through multiple binding sites.

**Fig. 4.**
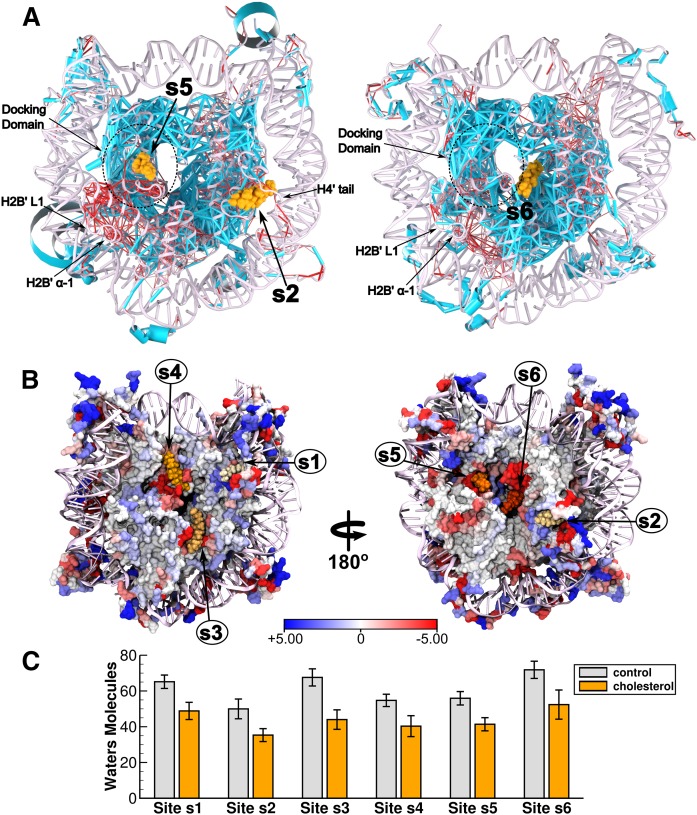
Position correlation analysis and hydration properties of the nucleosome. A: Shown are the cholesterol-induced Cα-Cα position correlation changes of the nucleosome. Edge thickness distinguishes between enhanced (light blue) and weakened (red) correlation changes due to cholesterol binding. By binding at sites s2, s5, and s6, note that cholesterol reduces coupling of specific regions nearby the docking domain and the H4 tail in both simulation systems. Here, Cα-Cα correlation values were calculated with Carma (35). B: Cholesterol-induced changes on the net number of water molecules bound to NCP residues within a cut-off distance of 3.5 Å. C: Average number of water molecules occupying each of the binding sites within a cut-off distance of 10 Å. Averages and associated standard errors were computed from 200 ns of simulation.

Within this scenario, we speculate that the observed macroscopic effects on chromatin structure and stability might result from dewetting of the nucleosome, triggered by cholesterol binding. Specifically, dewetting transitions may impact core-to-core interactions at hydrophobic regions, including the docking domain and the H3-H3 four helix bundle, with global impact on chromatin. Consistent with this hypothesis, we found that cholesterol dehydrates the nucleosome surface in ∼17 (±3) water molecules per binding site (**Fig. 4B, C**). As such, bound cholesterol reduces atom-to-atom position correlations at the docking domain and its neighboring regions, H2B loop 1 (L1) and helix 1 (α-1) (Fig. 4A), which likely influence critical packing interactions at the NCP. Note that in supplemental Fig. S4, this clear result also reflected on their increased root-mean-square fluctuations in the presence of bound cholesterol. Besides dewetting and packing effects, we also speculate that additional mechanisms accounting for chromatin modulation might exist. Specifically, cholesterol binding to the H4 tail, well-known to assist chromatin compaction by making contacts with the acidic patch of adjacent nucleosome units, may also interfere with the final chromatin state. Consistent with this notion, note that in Fig. 4A, the bound cholesterol reduces coupling between the nucleosome and the H4 tail, enhancing its mobility freedom (root-mean-square fluctuations) up to ∼2 Å, as compared with the ligand-free particle. Altogether, as shown in supplemental Fig., these views can be merged into a pictorial model that embodies the potential role of cholesterol in modulating particle-to-particle nucleosome interactions in chromatin.

It is known that some forms of transcriptionally silenced heterochromatin are localized adjacent to the nuclear lamina (31). Nevertheless, dynamically regulated genes have been found at the nuclear periphery when turned on (32). Despite that it is not clear how cholesterol under physiological conditions may interfere with chromatin structure inside the cell, together with previous evidence that it binds to chromatin (10), our findings suggest that cholesterol influences chromatin condensation by directly binding to the nucleosome.

Lipid microdomains, containing sphingomyelin and cholesterol in the inner nuclear membrane, have been suggested to provide a resting place for active chromatin and transcription factors (33). In addition, the tethering of chromatin to the nuclear periphery has an important role in the chromatin flow by defining changes in nuclear shape (34). We could raise the hypothesis that cholesterol might trigger chromatin structural changes that may contribute to the intra-nuclear organization of chromatin. It would be very interesting to precisely follow the changes in nuclear cholesterol circadian rhythm following the chromatin structural changes and nuclear localization, correlating them with the transcriptional activity.

## Supplementary Material

Supplemental Data
