# Supplementary material for: Biophysical studies of cholesterol effects on chromatin
Source: J Lipid Res. 2017 Apr 28;58(5):934–40. doi: 10.1194/jlr.M074997 (PMC5408612; doi:10.1194/jlr.M074997)
Supplement: Supplemental Data [file supp_58_5_934__index.html]

Biophysical studies of cholesterol effects on chromatin — Biophysical studies of cholesterol effects on chromatin — Supplemental Data 

# Biophysical studies of cholesterol effects on chromatin

## Supplemental Data

- Supplemental Material (.pdf, 5.1 MB) - Supplemental Material
