# Supplementary material for: Biophysical studies of cholesterol effects on chromatin
Source: J Lipid Res. 2017 Apr 28;58(5):934–40. doi: 10.1194/jlr.M074997 (PMC5408612; doi:10.1194/jlr.M074997)
Supplement: Supplemental Data [file 10.1194_M074997_jlr.M074997-1.pdf]

SUPPLEMENTAL INFORMATION:

Biophysical studies of cholesterol effects on chromatin

Isabel T. G. Silva☯[1], Vinícius Fernandes☯[1,2], Caio Souza[2], Werner Treptow*[2] & Guilherme M. Santos*[1]

1-Laboratório de Farmacologia Molecular, Departamento de Farmácia, Universidade de Brasília, Brasília, 70919-970 Brazil.

2- Laboratório de Biologia Teórica e Computacional, Departamento de Biologia Celular, Universidade de Brasília, DF 70910-900, Brasília, Brazil

☯ These authors contributed equally to this work.

Supplementary Methods

Negative staining Electron Microscopy of long chromatin fibers

The reconstituted chromatin fibers were prepared as in ref.(1). Briefly, a sample drop of 3μL was applied to an ultrathin carbon film 400-mesh copper grid (Ted Pella. Inc) previously treated by airglow discharge using an easiGlow discharge system (Pelco) with 15 mA positive current for 25 s in air atmosphere. After three minutes the sample was dried out from the grid using a filter-paper and a drop of uranyl acetate (2%) was added 3 times for 30 seconds each time, followed by blotting away the excess of staining. Specimens were analysed using a Jeol JEM-2100 operating at 200 kV. Images were acquired close to focus using an F-416 camera (TVIPS, Germany). Sample preparation and data acquisition were performed at the Electron Microscopy Laboratory – LME/LNNano.

Computational Methods

An atomistic model of the nucleosome core particle (NCP) was built using it's high-resolution x-ray, PDB code 1KX5 (2). The VMD software (3) was used to solvate with TIP3 waters (4) and neutralize with counter ions the NCP, resulting in a simulation cell with 152 Å x 225 Å x 100 Å, ~230.000 atoms and 150 mM sodium chloride. This system was thermalized and subsequently equilibrated in ~ 20 ns MD simulation. All simulations were run by NAMD version 2.10 (5) with CHARMM 36 force field (6) parameters updated for the cholesterol (7). PME method (8) was employed on the electrostatic calculations and non-bonded interactions were cut-off at 11 Å. All systems were simulated in a NPT ensemble at 300 K, 1 atm and with 2 fs time step.

We probed the cholesterol binding to NCP using the software AutoDock Vina (9). The ligands atomic coordinate was gathered in the PDB database with the accession code 1N83 (10).To acccount for the receptor rigidity imposed by Vina, cholesterol was docked to 120 independent NCP structures randomly collected from the last 6 ns

of equilibration. Ligands were allowed to have flexible bonds. Docking search took place in a 110 Å x 110 Å x 110 Å box comprising the complete NCP except the histone tails that moved away from the nucleosome. The exhaustiveness value was set to 200 and the best solutions distributed over the protein-nucleosome interface were gathered from each docking calculation, resulting in approximately 5000 solutions that were clustered in 20 groups based on a maximum neighbourhood criterion. Six of these groups were at sites that involve amino acids well-known to stabilize the nucleosome structure. The best solution from each of these six groups was included in the equilibrated NCP system. Cholesterol molecules were divided in two 200ns-long simulations, in which the minimum distance between ligands were 31 Å, avoiding any interference between them. Thus, cholesterol molecules at sites s2, s3 and s5 were included in one independent simulation and sites s1, s4 and s6 in another. No bias was needed to keep the cholesterol molecules bound to NCP throughout the entire simulation.

The Linear Interaction Energy method was employed in the free energy calculations presented in this article. This method states that the binding free energy of a ligand to a receptor is given by (11):

$$\Delta G^{o}_{bind} = \alpha\left(\langle V^{vdW}\rangle_{bound} - \langle V^{vdW}\rangle_{free}\right) + \beta\left(\langle V^{elect}\rangle_{bound} - \langle V^{elect}\rangle_{free}\right) + \gamma \quad [1]$$

where, $\langle V^{vdW}\rangle$ and $\langle V^{elect}\rangle$ are ensemble averages of the van der Waals and electrostatic interaction potentials of the ligand, when bound to the receptor or free in solution. The parameters α and β are empirical scaling factors (here set to 0.18 and 0.5), whereas γ is a correction term (here set to 0). Importantly, a value of $\alpha = 0.18$ was parameterized using experimental binding free energies and thus takes into account all van der Waals contributions to the ligand binding, e.g., rotational and translational entropies (12). The non-bonded interaction potentials V^{X} are calculated directly from the microscopic configurations of the system generated through MD simulations. Here, $\langle V^{vdW}\rangle_{bound}$ and $\langle V^{elect}\rangle_{bound}$ were computed from the simulation with cholesterol bound to NCP (Supplemental Figure S6). $\langle V^{vdW}\rangle_{free}$ and $\langle V^{elect}\rangle_{free}$ were sampled from a 200ns-long simulation of one cholesterol in pure water. The binding constant (K) was then calculated as:

$$K = \frac{e^{\frac{-\Delta G}{k_b T}}}{C^o} [2]$$

where k_b, T and C^o are respectively the Boltzmann constant, temperature and standard concentration.

The network dynamical analysis allows investigating the interaction correlation between pairs of nodes, which in our analysis were represented by the Cα atom of each amino acid. Each pair of node is connected by an edge, which thickness are weighted by their correlation value. The correlation was calculated here using the program Carma (13).

Supplementary Tables

Supplemental Table S1. Residues less than 3.5 Å from cholesterol at selected sites.

Sites	H3/H3'	H4/H4'	H2A°/H2A'°	H2B/H2B'*
s1		**H18, R19, K20,** Q27		
s2	L65', Q68', R69', R72', Q76'	**H18',** L22', **R23'**		
s3	**F78**	R67		**R96**, G101, A104, **K105**, V108
s4	I112', H113', Q125, R128, R129, R134		V114	
s5	A98, V101, A102		R88', **E91'**, N94', G98', T101', G106', V107', L108', N110'	
s6	G132', **E133'**	Q93', G94', R95'	*E92', K95', L96'	P100'

Bold are NCP amino acids involved on binding of NBPs(15). Amino acids 80 to 119 are from the docking domain. The symbol ' differentiate between histones copies

Supplementary Figures

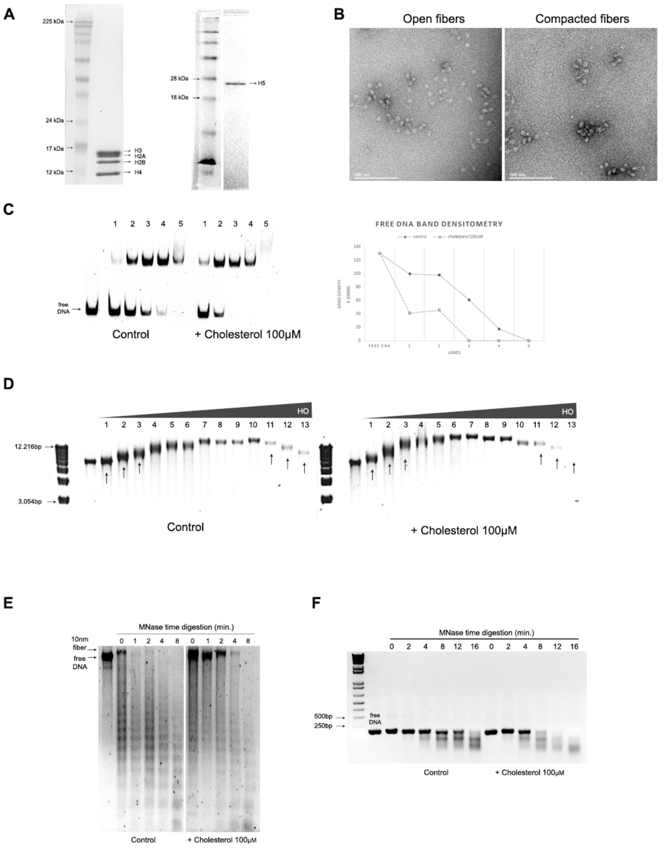

Supplemental Figure S1. In vitro analysis. a) Histone octamer and H5 integrity. Denaturing acrylamide gel 15% stained with Comassie blue; b) Electron microscopy images of negative staining chromatin fibers reconstituted in vitro. Array: 177.36; c) Histone octamer titration (1= 0.5; 2=1.0; 3= 1.6; 4= 2.0; 5= 2.7µM) in absence (control) or presence of cholesterol 100µM, analysed by EMSA in agarose gel 0.8%, Array: 167.1. Gels run under the same experimental conditions. Free DNA band densitometry graph of left gels; d) Histone octamer titration (1= 0.45; 2= 0.68; 3= 0.90; 4= 1.1; 5= 1.3; 6= 1.5; 7= 1.8; 8= 2.0; 9= 2.2; 10= 2.5; 11= 2.7; 12= 2.9; 13= 3.4µM) in absence (control) or presence of cholesterol 100µM, analysed by EMSA in agarose gel 0.8%, Array: 177.36. Gels run under the same experimental conditions. Arrows indicates prominent differences of point saturation in presence of cholesterol; e) MNase digestion of 10nm fibers in presence or absence of 100µM cholesterol. Agarose gel 0.8%. Array: 177.36; f) MNase digestion of naked DNA (167.1) in presence or absence of 100µM cholesterol. Agarose gel 0.8%.

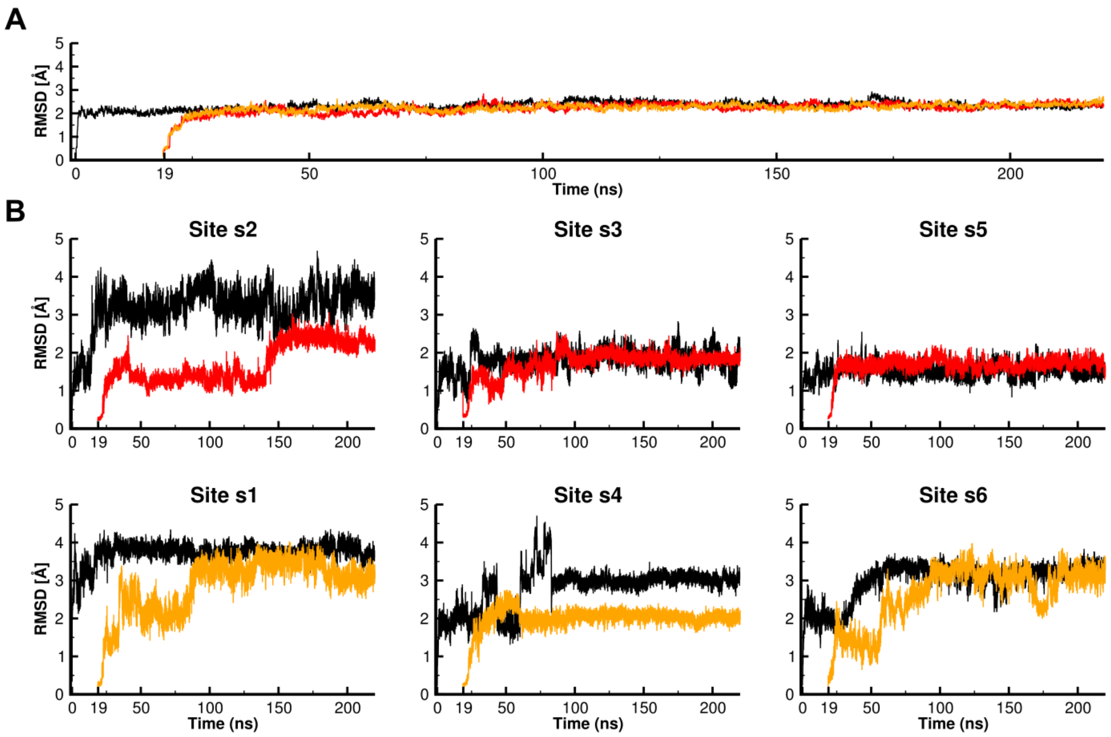

Supplemental Figure S2. All-atom root mean square deviation (RMSD) profiles for free- (black) and bound-cholesterol (red and orange) systems. a) NCP histones; b) Binding sites. RMSDs for the binding sites were computed by taking into consideration the reference amino acids shown in the Table S1.

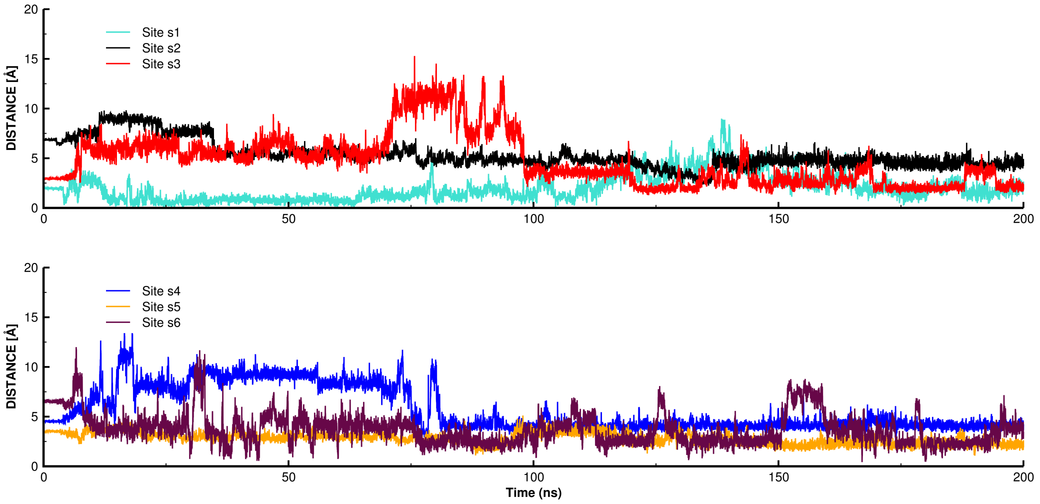

Supplemental Figure S3. Shown are profiles reporting the time dependent centroid distances between the cholesterol and each of the binding sites. Centroid positions for the binding sites were computed from the reference amino acids shown in the Table S1.

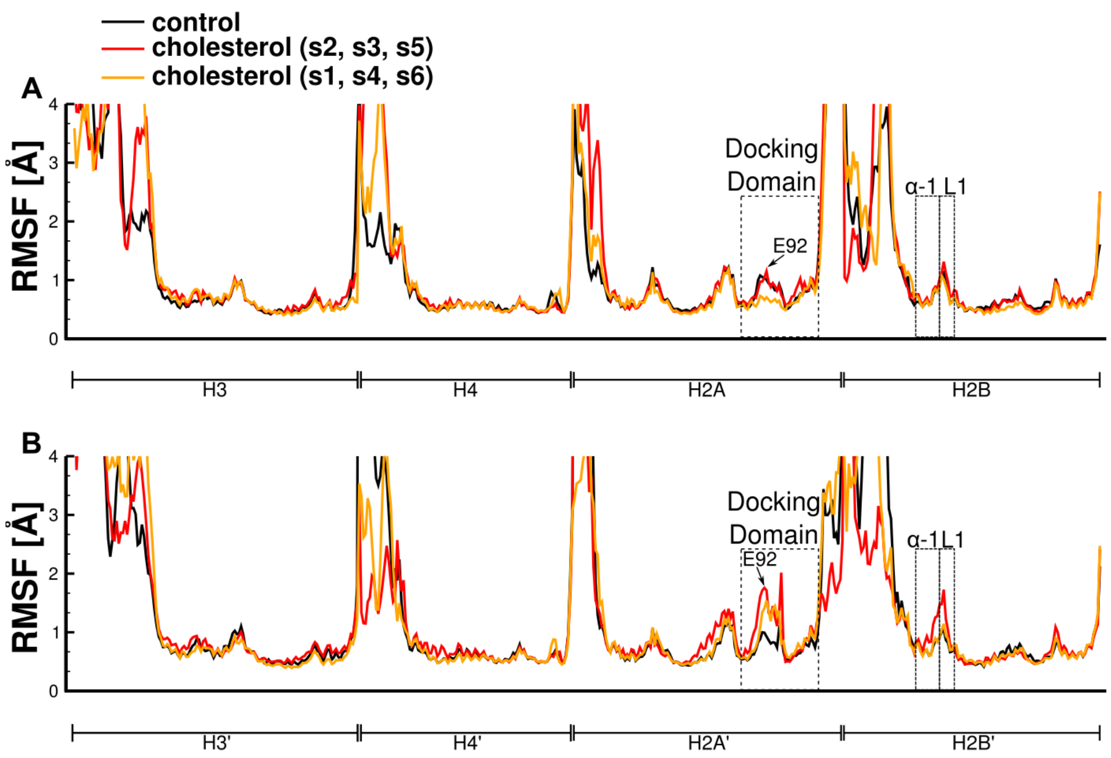

Supplemental Figure S4. Root mean square fluctuation (RMSF) profiles for free- (black) and bound-cholesterol (red and orange) systems. Profiles are shown separately for the Cα-atoms of histones (a) H3, H4, H2A and H2B, and (b) H3', H4', H2A' and H2B'. RMSF values larger than 4 Å are not shown for clarity. Note that bound cholesterol impacts significantly on the atomic fluctuations of the docking domain and its neighbouring regions, H2B loop 1 (L1) and helix 1 (α-1).

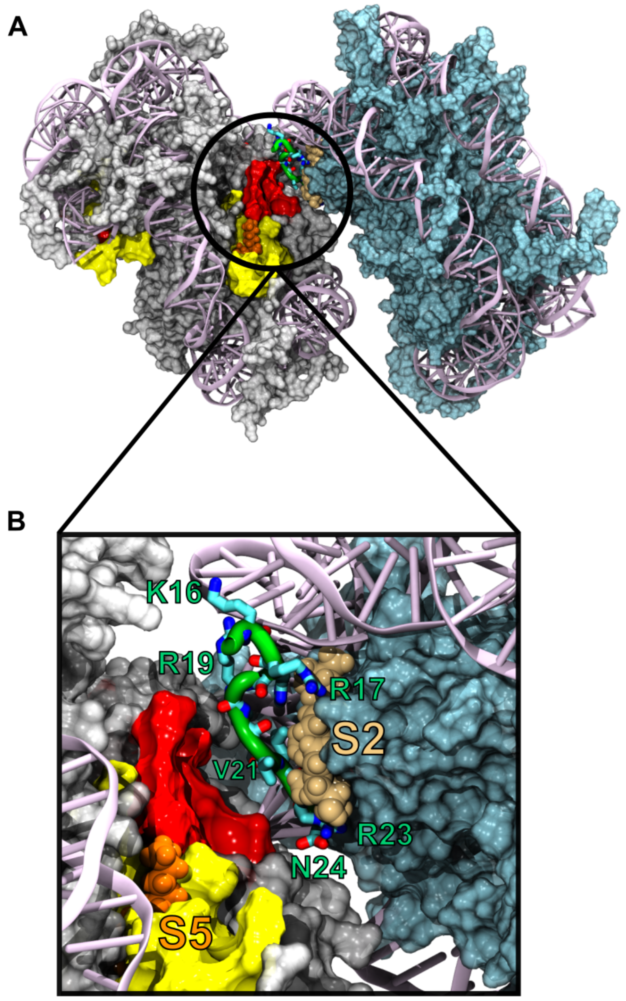

Supplemental Figure S5. Pictorial model for the potential role of cholesterol in modulating particle-to-particle nucleosome interactions in chromatin. a) Shown are two nucleosome units (blue and silver) highlighting putative interactions of the H4 tail (green) at the acidic patch region (red), and the presence of cholesterol at the docking domain (yellow); b) Close view of (a), showing key amino acids for H4-tail acidic-patch interactions (K16, R17, R19, V21, R23). This model was inspired on that devised by Dhal and colleagues (14).

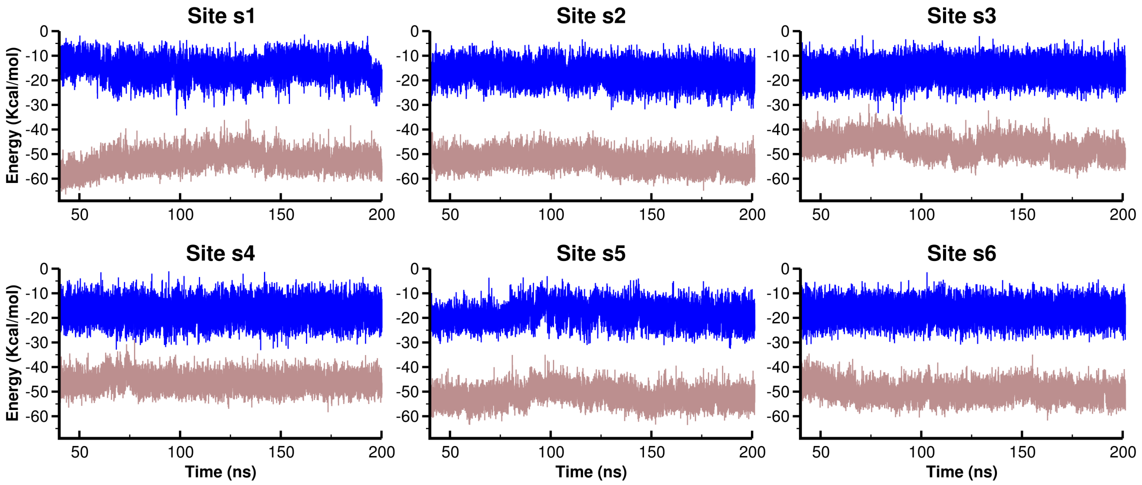

Supplemental Figure S6. Per site van der Waals (brown) and Coulomb (blue) interaction energies for bound cholesterol throughout the simulation.

Supporting References

1. Huynh, V. a T., P. J. J. Robinson, and D. Rhodes. 2005. A method for the in vitro reconstitution of a defined "30 nm" chromatin fibre containing stoichiometric amounts of the linker histone. *J. Mol. Biol.* **345**: 957–968.

2. Davey, C. a, D. F. Sargent, K. Luger, A. W. Maeder, and T. J. Richmond. 2002. Solvent mediated interactions in the structure of the nucleosome core particle at 1.9 A resolution. *J. Mol. Biol.* **319**: 1097–1113.

3. Humphrey, W., A. Dalke, and K. Schulten. 1996. VMD: Visual Molecular Dynamics. *J. Mol. Graph.* **14**: 33–38.

4. Jorgensen, W. L., J. Chandrasekhar, J. D. Madura, R. W. Impey, and M. L. Klein. 1983. Comparison of simple potential functions for simulating liquid water. *J. Chem. Phys.* **79**: 926–935.

5. James C. Phillips, R. Braun, W. Wang, J. Gumbart, E. Tajkhorshid, E. Villa, C. Chipot, R. D. Skeel, L. Kalé, and K. Schulten. 2005. Scalable Molecular Dynamics with NAMD. *J Comput Chem*. **26**: 1781–1802.

6. Huang, J., and A. D. J. MacKerell. 2014. CHARMM36 all-atom additive protein force field: Validation based on comparison to NMR data. *J. Comput. Chem.* **34**: 2135–2145.

7. Lim, J. B., B. Rogaski, and J. B. Klauda. 2012. Update of the cholesterol force field parameters in CHARMM. *J. Phys. Chem. B*. **116**: 203–210.

8. Darden, T., D. York, and L. Pedersen. 1993. Particle mesh Ewald: An N.log(N) method for Ewald sums in large systems. *J. Chem. Phys.* **98**: 10089–10092.

9. Trott, O., and A. J. Olson. 2010. AutoDock Vina: Improving the speed and accuracy of docking with a new scoring function, efficient optimization, and multithreading. *J. Comput. Chem.* **31**: 455–461.

10. Kallen, J. a., J.-M. Schlaeppi, F. Bitsch, S. Geisse, M. Geiser, I. Delhon, and B. Fournier. 2002. X-Ray Structure of the hRORα LBD at 1.63 Å: Structural and Functional Data that Cholesterol or a Cholesterol Derivative Is the Natural Ligand of RORα. *Structure*. **10**: 1697–1707.

11. Åqvist, J., C. Medina, and J.-E. Samuelsson. 1994. A new method for predicting binding affinity in computer-aided drug design. *Protein Eng.* . **7** : 385–391.

12. Carlsson, J., and J. Aqvist. 2006. Calculations of solute and solvent entropies from molecular dynamics simulations. *Phys. Chem. Chem. Phys.* **8**: 5385–5395.

13. Glykos, N. M.. 2006. Software News and Updates Carma: AMolecular Dynamics Analysis Program. *J. Comput. Chem.* **27**: 1765–1768.

14. Dhall, A., S. Wei, B. Fierz, C. L. Woodcock, T. H. Lee, and C. Chatterjee. 2014. Sumoylated human histone H4 prevents chromatin compaction by inhibiting long-range internucleosomal interactions. *J. Biol. Chem.* **289**: 33827–33837.

15. Silva, I. T. G. da, P. S. L. de Oliveira, and G. M. Santos. 2015. Featuring the nucleosome surface as a therapeutic target. *Trends Pharmacol. Sci.* **36**: 263–269.
